# Effects of Dietary Guanidinoacetic Acid on the Performance, Rumen Fermentation, Metabolism, and Meat of Confined Steers

**DOI:** 10.3390/ani14172617

**Published:** 2024-09-09

**Authors:** Gabrielly Chechi Giraldi, Gabriel Jean Wolschick, Mateus Henrique Signor, Rafael Vinicius Pansera Lago, Ana Luiza de Souza Muniz, Taynara Monica Reginatto Draszevski, Manoela Meira Balzan, Roger Wagner, Aleksandro Schafer da Silva

**Affiliations:** 1 Graduate Program in Animal Science, Universidade do Estado de Santa Catarina (UDESC), Chapecó 89815-630, SC, Brazil; gabrielly.giraldi@edu.udesc.br (G.C.G.); mateus.signor@edu.udesc.br (M.H.S.); rafael.lago@edu.udesc.br (R.V.P.L.); 2 Department of Animal Science, Universidade do Estado de Santa Catarina (UDESC), Chapecó 89815-630, SC, Brazil; gabriel.jw@edu.udesc.br (G.J.W.); ana.lms20@edu.udesc.br (A.L.d.S.M.); taynara.draszevski@edu.udesc.br (T.M.R.D.); 3 Department of Food Science, Universidade Federal de Santa Maria, Santa Maria 97105-900, RS, Brazil; manoela.balzan@acad.ufsm.br (M.M.B.); rogerwag@gmail.com (R.W.)

**Keywords:** guanidinoacetic acid, beef, creatine, performance, bovine

## Abstract

**Simple Summary:**

Guanidinoacetic acid (GAA) is a natural derivative of amino acids. They are acted upon by enzymes in the liver and transformed into essential components of the energy metabolism of muscle and nervous tissue. Although the animal synthesizes creatine, the amount is less than necessary for high growth rates, according to researchers. GAA consumed by cattle reduces fat between muscle fibers and serum cholesterol. It has a mild but positive effect on the fatty acid profile of meat—i.e., it increases myristoleic, linoleic, and arachidonic acids. We also concluded that GAA consumption increases the amount of volatile fatty acids in the rumen, especially acetic, propionic, and butyric acid. We draw attention to the acetic acid related to lipid synthesis.

**Abstract:**

With the increase in population, it is increasingly necessary to produce food more efficiently. This has expanded the market for additives, which are products that directly (nutritional effect) or indirectly (effect on animal health) favor productivity. Guanidinoacetic acid (GAA) is a natural precursor of creatine. It acts as an energy reserve in skeletal muscle. In addition to being a compound with more significant bioavailability, it is more thermally stable and less expensive than creatine. Therefore, this study aimed to determine whether adding GAA to the cattle diet would alter the meat’s composition and fatty acid profile. We used 24 Holstein cattle males (409 ± 5.6 kg), approximately 15 months old, and separated them into four homogeneous groups, one being the control group and three groups with various dosages of GAA in the diets (3.3; 6.6, and 9.9 g/animal/day), for an experimental period of 60 days. Blood, rumen fluid, and animal weighing were performed at three points (days 1, 30, and 60), and daily feed consumption was measured. Steers fed with GAA (9.9 g/d) showed a 16.9% increase in average daily gain (ADG) compared to the control group. These same animals (T-9.9 group) fed with GAA showed a 20% increase in fed efficiency compared to the control group. Lower leukocyte, lymphocyte, and granulocyte counts and lower cholesterol levels were observed in animals that consumed 6.6 g and 9.9 g/d GAA compared to the control group. Animals from the T-6.6 and T-9.9 groups showed 30% and 27.6% reduced bacterial activity in the rumen compared to the control group, respectively. Steers from the T-6.6 and T-9.9 groups fed with GAA showed a 20% and 37% increase in short-chain fatty acids (SCFAs) compared to the control group, respectively. A higher concentration of acetic, propionic, and butyric acids in the ruminal fluid of cattle T-9.9 group was observed at day 60. The two highest doses of GAA showed lower fat levels in the meat, just as the cattle that received 9.9 g/d showed higher levels of total polyunsaturated fatty acids. Complementary data results draw attention to the dose of 9.9 g/d GAA in cattle diets, as anti-inflammatory action can be seen and combined with a higher concentration of SCFAs, consequently increases weight gain. We concluded that consuming this GAA increases the concentration of some unsaturated fatty acids (omegas) in the meat, which adds quality to the product for the consumer.

## 1. Introduction

Efficient food production is increasingly necessary due to population growth [1], but mainly because production costs are high. The diet is primarily responsible for productive efficiency as it provides animals with all the nutrients necessary for their growth, maintenance, and production [1]. However, it is already known that additives can enhance the animal’s use of this diet. This includes guanidinoacetic acid (GAA), which, when added to the diet of beef cattle, enhanced productive performance [2].

GAA is a natural derivative of amino acids, which are acted upon by enzymes in the liver and transform into an essential component in the energy metabolism of muscle and nervous tissue [3]. Although the animal synthesizes creatine, the amount is less than necessary for high growth rates according to researchers [4].

GAA synthesis occurs in the kidney, where the amino acid arginine methyl is transferred to the amino acid glycine via the enzyme l-arginine-glycine amidinotransferase (AGAT). Upon receiving this amino group, glycine becomes GAA, and ornithine, an amino acid, sends negative feedback to the AGAT enzyme, decreasing GAA synthesis [4]. When we provide GAA in the diet, its synthesis does not occur, resulting in the saving of arginine, which is an essential amino acid for protein synthesis, hormonal secretion, and cell signaling [3]. According to Ostojic [3], the supply of GAA via the diet can also improve antioxidant status, increase antioxidant and energetic capacity, act as a vasodilator, and stimulate insulin and glucagon.

GAA has more bioavailability than creatine because it has more transporters [5], and supplemented GAA elevates hepatic and muscular creatine [6]. GAA has good thermal stability [4] and is more affordable than creatine. Although there are studies on cattle in terms of performance, oxidative systems, and ruminal fermentation [2,7,8], little is known about the effect of GAA on beef related to the fatty acid profile. In broiler chickens, GAA led to lower pH and luminosity in meat and increased GAA and creatine [9]. A recent study used in vitro testing to show that GAA alters the abundance and diversity of microorganisms in the rumen [10]. Therefore, we hypothesize that this change can alter the profile of SCFAs, which could influence beef. Hence, this study aimed to determine whether adding GAA to the diet of Holstein steers would influence the meat’s chemical composition and fatty acid profile.

## 2. Materials and Methods

### 2.1. Ethics

The Ethics Committee on the Use of Animals in Research (CEUA—UDESC) approved the project under protocol number 2674260323.

### 2.2. Test Product

The GAA (C_3_H_7_N_3_O_2_) used in this experiment was purchased from Nutriquest and sold commercially as Creamino^®^ (Alzchem GmbH, Trostberg, Alemanha)—the purity was at least 96% for GAA with a maximum of 1% water. Creamino^®^ is a white to slightly beige granulated powder, with a bulk density of 600 kg/m^3^ and solubility of approx. 4.0 g/L water at 20 °C.

### 2.3. Animals and Installations

The experiment was conducted at the UDESC Oeste Experimental Farm (FECEO) in the ruminants sector and lasted two months. We used 24 Holstein cattle males (409 ± 5.6 kg), approximately 15 months old and housed in individual stalls with feeders and drinkers. The experiment occurred during the Brazilian winter (July to September) when temperature and humidity fluctuated during the day and the experimental period. Temperatures during the experimental period ranged between 3.2 °C and 26.8 °C, with relative humidity between 65 and 92%. The lighting for the animals was natural, with a period of darkness during the night.

### 2.4. Experimental Design, Diets and Performance

The animals were divided into as follows: Treatment T-0 (*n* = 6), which received the basal diet; Treatment T-3.3 (*n* = 6), feed with the addition of 3.3 g product/animal/day; Treatment T-6.6 (*n* = 6), feed with the addition of 6.6 g product/animal/day; Treatment T-9.9 (*n* = 6), feed with the addition of 9.9 g product/animal/day. The formulation of the diet aimed to meet all the nutritional requirements (Table 1) of the animals of Brcorte [11] and was supplied in the morning and afternoon, with monitoring and quantifying of leftovers. Samples of feed provided were collected throughout the experiment, and at the end, the total diets were pooled for chemical composition analysis.

### 2.5. Data and Sample Collection

In the morning, approximately 13 ± 0.5 h after the last feeding of the previous day, the animals were restrained for weighing and sample collection (blood and ruminal liquid). The cattle were weighed on days 1, 30, and 60 of the experiment using a digital scale. Weight gain (WG) and average daily weight gain (DWG) were calculated based on the weighing results. Daily feed intake (DFI) was measured individually for the cattle. Using this data, feed efficiency was calculated: DWG/DFI.

During the experimental period, three blood collections were carried out (days 1, 30, and 60). The collection was performed through the coccygeal vein, with the aid of needles and vacuolated tubes, without reagent to obtain serum for biochemical analyses, oxidant and antioxidant levels, and using vacuolated tubes with anticoagulant (EDTA) for hematologic analysis. After collection, the tubes were placed in thermal boxes with recyclable ice for refrigeration. Serum separation was undertaken by centrifuging the tubes without anticoagulant at 820 gravity (g) for 10 min. The serum was transferred to microtubes and stored at −20 °C until analysis.

On days 1, 30, and 60, 200 mL of rumen liquid was collected using a 1.5 m oro-ruminal tube with an 11 mm diameter four hours after treatment. After collection, the pH was read using a portable digital pH meter, model AK103, and the functional activity of the ruminal microbiota was measured using the methylene blue reduction test (MBRT) as described by Dirksen and Smith [12]. The remaining liquid was filtered through triple layers of gauze and stored in 5 mL microtubes for volatile fatty acid (VFA) analysis. Samples of VFA were stored at −80 °C until analysis.

After the end of the experimental period (day 61), the animals were slaughtered in a commercial slaughterhouse, where the slaughter took place following the regulations in force in Brazil and was accompanied by a veterinarian from the federal inspection sector. Slaughter was carried out using a pneumatic pistol to stun followed by bleeding, with these procedures being the responsibility of the slaughterhouse. At the end of the slaughter line, after weighing and before the carcass went to the cold room, the left longissimus dorsi muscle was collected for further analysis. The muscle was placed in a plastic bag, identified, and stored in a thermal box with ice during collection and transport. Upon arriving at the laboratory, the muscle was placed in the refrigerator for 20 h (5 °C) to await the rigor of death. Then, 24 h after slaughter, meat analysis began, and slices of meat were separated and frozen (−20 °C).

### 2.6. Laboratory Analysis

#### 2.6.1. Feed Analysis

In the laboratory, the feed samples were pre-dried in a forced ventilation oven at 55 °C for 72 h, then removed from the oven and weighed again to determine the partial dry matter content, and followed by grinding in a Wiley mill (Marconi, model: MA340, Piracicaba, SP, Brazil) using a 1 mm mesh sieve. The pre-dried and ground samples were heated at 105 °C to obtain the DM and the mineral material in a muffle furnace at 600 °C [13]. The micro-Kjeldahl method determined the N content (Method 984.13) [14], which made it possible to predict the CP content through mathematical calculation. To determine the neutral detergent fiber content, the samples were placed in polyester bags [15] and treated with a neutral detergent solution in an autoclave at 110 °C for 40 min [16]. For concentrate samples, we included α-amylase [17]. Acid detergent fiber concentrations were determined according to AOAC [13] (method 973.18). The values of starch, carbohydrates, and ether extract were obtained using a Near Infrared Reflectance Spectrometer (NIRS), model 12 Spectra Star 2600 XT series of Near Infrared Analyzers (Unity Scientific^®^, Malvern, UK). The results are presented in Table 1.

**Table 1:** Chemical composition and fatty acid profiles of the total diets (TMR) available to cattle.

Variables	T-0	T-3.3	T-6.6	T-9.9
Dry matter, %	47.2	47.2	48.2	48.7
Mineral matter, %	6.09	5.80	6.69	6.15
Crude protein, %	13.4	13.8	12.9	12.9
NDF, %	31.5	29.9	31.1	31.3
ADF, %	14.3	13.0	14.4	14.4
Etherel extract, %	3.79	4.13	3.99	4.07
Fatty acid, %				
C6:0 (Caproic)	0.06	0.02	0.02	0.02
C8:0 (Caprylic)	0.04	0.02	0.02	0.02
C10:0 (Capric)	0.01	0.02	0.01	0.01
C12:0 (Lauric)	0.29	0.27	0.31	0.25
C14:0 (Myristic)	0.19	0.16	0.16	0.17
C15:0 (Pentadecanoic)	0.16	0.11	0.10	0.12
C16:0 (Palmitic)	17.5	16.1	16.9	15.8
C17:0 (Heptadecanoic)	0.21	0.18	0.18	0.19
C17:1 (cis-10-Heptadecenoic)	0.05	0.05	0.05	0.05
C18:0 (Stearic)	3.58	2.91	3.07	3.16
C18:1n9t (Elaidic)	0.08	0.06	0.07	0.07
C18:1n9c (Oleic)	25.3	25.8	27.4	27.3
C18:2n6c (Linoleic)	45.5	48.1	45.9	46.6
C20:0 (Arachidic)	0.48	0.48	0.52	0.51
C20:1n9 (cis-11-Eicosenoic)	0.29	0.30	0.32	0.30
C18:3n3 (a-Linolenic)	3.79	3.83	3.53	3.68
C20:2 (cis-11,14-Eicosadienoic)	0.24	0.14	0.12	0.25
C22:0 (Behenic)	0.46	0.44	0.45	0.48
C22:1n9 (Erucic)	0.10	0.10	0.10	0.13
C23:0 (Tricosanoic)	0.11	0.11	0.11	0.11
C24:0 (Lignoceric)	0.50	0.51	0.53	0.54

Note: The TMR was formulated based on corn silage (40%), ground corn (30%), wheat bran (15%), soy hulls (6.76%), soy bran (5.64%), and mineral supplement (2.6%): calcium 170–190 g/kg, phosphorus 40 g/kg, sodium 133 g/kg, magnesium 5 g/kg, sulfur 6 g/kg, zinc 2000 mg/kg, copper 600 mg/kg, iron 400 mg/kg, manganese 500 mg/kg, cobalt 40 mg/kg, iodine 35 mg/kg, selenium 9 mg/kg, and fluorine max. 400 mg/kg (Bovicort 40^®^, Nuctramix, Chapecó, Brazil).

#### 2.6.2. Complete Blood Count (CBC)

After collection, hemoglobin, erythrocytes, leukocytes, hematocrit, and leukocyte differentials were determined using an Equip Vet 3000 3-part automatic hematologic analyzer (Equip Diagnostica, Itatiba, SP, Brazil).

#### 2.6.3. Serum Biochemistries

Biochemical analyses were performed using a Bio-2000 BioPlus^®^ (Barueri, SP, Brazil) semi-automatic analyzer and commercial kits (Analisa^®^, Belo Horizonte, MG, Brazil), where the levels of total protein (PT), albumin, cholesterol, and urea and the enzymes aspartate aminotransferase (AST) and gamma-glutamyl transferase (GGT) were mensured. Globulin levels were obtained through mathematical calculations (total proteins—albumin).

#### 2.6.4. Meat Oxidative Status

To check the antioxidant/oxidative status, 0.5 g of the central area of the meat was removed and homogenized in a saline solution, the samples were centrifuged for 10 min at 7500× *g*, and the supernatants were stored in microtubes at −20 °C until analysis. Lipid extraction was performed according to Bligh and Dyer [18] with modifications; 1.5 g of the sample, 0.5 mL of distilled water, 0.5 mL of methanol, and 2.5 mL of chloroform were added to 15 mL polypropylene tubes and homogenized for 30 min, after which a solution of 2.5 mL of chloroform and 1.5% Na_2_SO_4_ were added to promote the two-phase system. The mixture was homogenized for 2 min and centrifuged for 15 min at 720× *g*. The lipids obtained in the chloroform phase were analyzed using fatty acids. Meat glutathione S-transferase (GST) was measured based on the methodology of Habig et al. [19] and expressed as µmol CDNB/min/mg of meat protein. Serum lipid peroxidation was measured based on the amount of thiobarbituric acid reactive substance (TBARS) according to Ohkawa et al. [20], and the results expressed in nmol MDA/mL. The methodology of Ellman [21] was used for total thiols, and the results are expressed as nmol SH/mg protein.

#### 2.6.5. Protein in Meat

A NIRS model Spectra Star 2600 XT series of Near Infrared Analyzers (Unity Scientific^®^, Malvern, UK) was used for meat protein values.

#### 2.6.6. pH, Color, and Retention Capacity

The pH of the hot carcass was measured using a portable digital pH meter after slaughter and 24 h after slaughter in meat samples at random points. Color evaluation was performed using a CR-400 chromometer (Minolka, Osaka, Japan), CIE1976 L* a* b*, and a D65 standard illuminant and compared to a CIE 1931 standard observer (x2λ, y λ, z λ) and a calibration plate (number 1849-701). Parameters were defined as brightness (L*), dark red (a*), and yellow (b*) and measured at three random sample points. The methodology adapted from Honikel [22] was used to determine the water retention capacity. A 0.5 g meat sample was weighed and positioned above a paper filter (15 × 15 cm) between two acrylic dishes. A 2 kg weight was placed above the sample for 5 min. After the sample was weighed again, the water loss calculation was made using the weight difference. The result is presented as a percentage.

#### 2.6.7. SCFA Profile in the Rumen

Rumen fluid samples were thawed at 5 °C and manually shaken for homogenization. Aliquots of 1 mL of the supernatant from the rumen fluid samples were collected in polypropylene microtubes (2 mL) and centrifuged for 5 min (12,300× *g*). After that, 250 μL of the supernatant was removed and transferred to a new microtube containing 250 μL of formic acid. The mixture was manually shaken and centrifuged again for 3 min. Then, 250 μL of the mixture supernatant was collected in an injection vial and added with 500 μL of 3-octanol solution (665 μg mL^−1^ in methanol) used as an internal standard and homogenized. Samples were analyzed on a gas chromatograph with a flame ionization detector (GC-FID; Varian Star 3600, Palo Alto, CA, USA) and an autosampler (Varian 8200CX, Palo Alto, CA, USA). We injected 1 μL of the extract in split mode at 1:10. The carrier gas used was hydrogen at a constant pressure of 20 psi. The analytes (acetic, propionic, butyric, valeric, and isovaleric acids) were separated by a CP-WAX 52CB capillary column (60 m × 0.25 mm; 0.25 μm stationary phase thickness). The initial column temperature was set at 80 °C for 1 min, increased to 120 °C at 8 °C min^−1^, and then to 230 °C at 15 °C min^−1^, where it remained for 1 min. The injector and detector temperatures were set at 250 °C. Method validation included the following parameters: selectivity, linearity, linear range, repeatability, precision, limit of detection (LOD), and limit of quantification (LOQ) for acetic, propionic, butyric, valeric, and isovaleric acids. Analytical parameters are presented in Supplementary Materials S1. Linearity was assessed by calculating a regression equation using the least squares method. LOD and LOQ values were achieved by sequential dilutions to signal-to-noise ratios of 3:1 and 6:1, respectively. Precision was assessed by analyzing the repeatability of six replicate samples. Accuracy was determined by recovering known amounts of standard substances added to a diluted sample. The results were expressed as mmol L^−1^ of each short-chain fatty acid (SCFA) in ruminal fluid.

#### 2.6.8. Fatty Acid Profile in Meat

To determine fatty acid methyl esters (EMAG), a gas chromatograph model, TRACE 1310, equipped with an ionized flame detector (Thermo Scientific, Waltham, MA, USA) was used. A total of 1 ml of samples was injected into a split/splitless injector, operated in split mode with a 1:20 ratio at 250 °C. Hydrogen was used as a transport gas in constant flow at 1.5 mL/min. FEMA separation was performed using an RT 2560 film (100 m × 0.25 mm × 0.20 µm thick, Restek, PA, USA) chromatographic column. The initial oven temperature was programmed at 100 °C for 5 min, increased to 180 °C at 8 °C/min, then increased at a flow rate of 4 °C/min until it reached 210 °C, and finally increased at 20 °C/min until it reached 250 °C; this temperature was maintained for 7 min. EMAG components were identified by comparing experimental retention times with authenticated standards (FAME Mix-37, Sigma Aldrich, St. Louis, MO, USA). The results were expressed as a percentage of each fatty acid identified in the lipid fraction, considering the EMAG chain equivalent factor for FID and the conversion factor for the respective acid, according to Visentainer and Franco [23].

### 2.7. Statistical Analyses

All data were analyzed using the SAS MIXED procedure (SAS Inst. Inc., Cary, NC, USA; version 9.4). Satterthwaite’s approximation determined the denominator degrees of freedom for the fixed effects test. WG, ADG, feed efficiency, and postmortem variables were tested for a fixed effect of treatment using animal (treatment) as a random effect. The remaining data were analyzed as repeated measures and tested for fixed effects of treatment, day and treatment × day, and using animal within each treatment as a random effect. The d 1 results were included as an independent covariate. Additionally, for these variables, the d1 scores were removed from the data set to generate the mean per treatment but were retained as covariates. The first-order autoregressive covariance structure was selected according to the lowest Akaike information criterion. Means were separated using the PDIFF method, and all results were reported as LSMEANS followed by standard error. Significance was defined when *p* ≤ 0.05 and trend when *p* > 0.05 and ≤0.10.

## 3. Results

The chemical composition and fatty acid profiles of the total diets (TMR) are presented in Table 1. The diets had approximately 13% crude protein.

### 3.1. Performance

The results related to performance are shown in Table 2. There was no treatment effect and no treatment × day interaction for body weight (*p* > 0.05). There was a difference in WG between days 1 and 60, where animals in T-9.9 showed more significant gain than the other treatments (*p* ≤ 0.10). There was also a difference in WG between days 15 and 60, in which the T-9.9 group showed more significant WG, followed by T-0, T-6.6, and T-9.9. The T-9.9 group also showed the highest ADG between days 15 and 60. There was no difference in feed conversion among the T-0, T-3.3, and T-6.6 groups, but the T-9.9 group showed the lowest feed conversion compared to the treatments. Also, this last group presented higher feed efficiency (T-9.9) than the others, which in turn did not differ from each other.

### 3.2. CBC and Serum Biochemistry

The results regarding the blood count are shown in Table 3. There was no treatment effect or treatment × day interaction for the parameters of monocytes, erythrocytes, hemoglobin, and hematocrits (*p* ≤ 0.05). The T-6.6 and T-9.9 groups showed lower leukocyte counts on days 30 and 60 than the control group; the same result was found for the lymphocyte and granulocyte counts. There was an effect of day on total leukocyte and lymphocyte counts in groups T-6.6 and T-9.9, being lower in both groups on days 30 and 60 compared to T-0. The other hematologic variables did not affect the day (Table 3).

The results regarding biochemical analyses are presented in Table 4. There was no treatment effect or treatment × day interaction for AST GGT enzymes and levels of total protein, albumin, globulin, and urea (*p* ≥ 0.10). There was an effect of treatment and treatment × day interaction (*p* ≤ 0.05) for cholesterol levels, where on days 30 and 60, the T-0 group presented higher levels than the others, followed by the T-3.3 group; the other groups were equal. There was no effect of day on clinical biochemistry variables in cattle (Table 4).

### 3.3. Ruminal Fluid

Regarding ruminal fluid analyses (Table 5), for the variables of pH, isobutyric, isovaleric, and valeric acid, there was no treatment effect and no treatment x day interaction (*p* ≥ 0.10). For MBRT, there was no treatment x day interaction (*p* ≥ 0.10). However, there was an effect of treatment, where the T-6.6 and T-9.9 groups showed more significant results than the other groups (*p* ≤ 0.05), thus obtaining lower bacterial activity. Concerning SCFAs, there was an effect of treatment and treatment x day interaction (*p* ≤ 0.05) on day 60, in which the T-9.9 group presented a higher count of total SCFAs, followed by the T-3.3, T-6.6, and T-0. A treatment effect and treatment x day interaction were observed for acetic acid on day 60 when animals in the T-6.6 and T-9.9 groups presented higher levels of acetic acid than T-0. Cattle from the T-1.5 g group had a higher concentration of propionic and butyric acids in rumen fluid than T-0 at day 60.

The day had an effect on SCFAs and acetic acid in the T-6.6 and T-9.9 groups (Table 5), being greater on day 60 than on day 30. The day had an effect on propionic and butyric acid in the T-9.9 group, being more significant on day 60 than on day 30. The other ruminal fluid variables did not affect the day.

**Table 5:** pH, bacterial activity, and short-chain fatty acid (SCFAs) profiles of Holstein cattle fed with guanidinoacetic acid additive.

Variables	T-0 ^1^	T-3.3	T-6.6	T-9.9	SEM	*p*-Val.: Treat ^1^	*p*-Val.: Treat × Day ^1^	*p*-Val.: Day ^2^
pH	6.41	6.30	6.40	6.25	0.03	0.53	0.42	0.63
MBRT (s)	72.6 ^b^	82 ^ab^	94.6 ^a^	92.7 ^a^	4.95	0.05	0.14	0.45
SCFAs (mmol/L)						0.01	0.01	0.01
d30	86.1	101	89.9 ^Y^	89.6 ^Y^	3.20			
d60	88.3 ^b^	100 ^ab^	106 ^aX^	121 ^aX^	3.14			
Acetic (mmol/L)						0.07	0.01	0.01
d30	58.9	68.9	60.7 ^Y^	58.9 ^Y^	3.04			
d60	61.8 ^b^	68.2 ^ab^	73.9 ^aX^	78.4 ^aX^	2.46			
Propionic (mmol/L)						0.05	0.01	0.01
d30	15.2	17.7	15.5	14.8 ^Y^	1.74			
d60	15.1 ^c^	17.9 ^bc^	19.4 ^b^	23.7 ^aX^	1.72			
Butyric (mmol/L)						0.04	0.01	0.05
d30	9.2	11.8	10.5	10.7 ^Y^	0.97			
d60	9.10 ^b^	11.3 ^ab^	11.6 ^ab^	15.6 ^aX^	0.92			
Isobutyric (mmol/L)	0.79	0.83	0.90	0.84	0.06	0.61	0.54	0.58
Isovaleric (mmol/L)	1.05	1.3	1.21	1.23	0.08	0.36	0.22	0.64
Valeric (mmol/L)	0.67	0.73	0.64	0.86	0.03	0.12	0.20	0.89

^1^ The treatments were as follows: T-0, basal diet; T-3.3, feed with the addition of 3.3 g of product/animal/day; T-6.6, feed with the addition of 6.6 g of product/animal/day; T-9.9, feed with the addition of 9.9 g of product/animal/day. ^1^ Within a line (a–c), they differ (*p* ≤ 0.05) between groups for the treatment effect and treatment × day interaction. ^2^ day effect: difference was illustrated by capital letters (X, Y) in the same column and by treatment in each variable.

### 3.4. Meat Analysis

The results related to meat quality can be found in Table 6. There was no treatment effect for the analyses of carcass yield, hot and cold carcass pH, luminosity, color, water retention capacity, protein, ash, TBARS, thiols, and GST analyses. Concerning fat, the T-3.3 and T-0 groups presented higher levels, followed by the T-6.6 and T-9.9 groups.

There was a difference in treatment for tetradecanoic acid (*p* < 0.05), where the T-9.9 and T-6.6 groups presented higher levels followed by the T-0 and T-3.3 (Table 7). There was a tendency for a treatment effect on linoleic acid values (*p* ≤ 0.10), where higher concentrations of this parameter were observed in the T-9.9 group compared to the control group (T-0). There was a tendency for treatment effect on linoleic acid values (*p* ≤ 0.10), where higher concentrations of this parameter were observed in the T-9.9 group compared to the control group (T-0). Animals treated with higher concentrations of guanidinoacetic acid (T-9.9) showed higher values of arachidonic acid than those in the control group (T-0). A difference was found for the sum of polyunsaturated fatty acids (PUFAs), where the T-9.9 group presented higher levels of PUFAs than the other groups.

## 4. Discussion

If expected at this stage, the animal will have an ADG of approximately 1.2 to 1.8 kg, depending on the diet provided [24]. Holstein steers in this study had a daily gain of around 1.36 kg. The group with the largest addition of GAA to the diet showed more significant WG, possibly because GGA is a precursor of creatine. It increases the amount of water in the cell as it is an osmotically active substance. Dangott et al. [25] showed that creatine supplementation can induce protein synthesis and decrease proteolysis, leading to an increase in lean mass and, consequently, increasing body weight.

Li et al. [2], when testing the inclusion of three different doses of AGG in the Angus diet, found that animals in the group with the addition of 0.9 g/kg dry matter obtained an ADG of 1.82 kg, while animals in the control group in turn obtained ADG of 1.42 kg. The group that received the highest dosage of GAA had lower FC, which indicates that the animal gained more efficient response. In a study carried out with broiler chickens, researchers showed that the addition of GAA in the finishing phase improved FC. The authors attributed this improvement to more significant muscle deposition and yield during the final phase [26]. The groups that showed better FC improved feed efficiency since the animals consumed less feed and gained more weight.

Blood comprises two groups of cells: red blood cells, which are red cells, and leukocytes, which are white cells responsible for defending against infections in the body. The increase in leukocytes in the blood can occur for several reasons, such as autoimmune diseases, viral infections, and nutritional deficiency, among many other factors. Although the groups that received the addition of GAA in the diet had lower white blood cell counts, no studies relate GAA to the immune system or inflammatory responses. Still, the leukocyte count can be lower when the organism is not challenged, and this means there is no stimulation for it, which occurs in animals with well-being and excellent health. In chickens, researchers verified a relationship between GAA consumption and oxidative stress [27], where the authors concluded that the performance of heat-stressed broilers fed by GAA is associated with increased muscle energy metabolism. This indirectly can also support tolerance against oxidative stress and consequently, the inflammatory response, which could explain the lower lymphocyte count.

Cholesterol produces cell membranes and some hormones, bile acids, and vitamin D [28]. The consumption of GAA by cattle affected cholesterol levels, being lower in animals with higher doses of GAA. When evaluating the cholesterol of cattle supplemented with different levels of GAA inclusion, there was no difference between the control and treatment groups [7]. In tilapia, researchers found that the fish that consumed GAA had higher cholesterol levels [29], which are results similar to our research. Recently, researchers found that the consumption of GAA by ducks reduced total cholesterol, malondialdehyde, triglycerides, and abdominal fat percentage, and creatine contents were increased in liver and breast muscles, which, according to the authors, is an indication that the GAA improved the lipid metabolism in ducks and changed the fat deposition and lipid levels in the liver [30]. However, it is challenging to compare the studies since they are species with different metabolisms. Future studies should clarify the mechanism involved.

For the fermentation and degradation of feed to occur correctly in the rumen, the pH must be between 6.0 and 7.0, allowing the microorganisms present in the rumen flora to proliferate correctly and avoiding metabolic disorders [24]. All experimental groups presented pH within the expected range. The MBRT determines the microbial activity in rumen fluid; typical values are between 3 and 6 min [30]. The groups that received the additive showed higher values for the reduction (consumption) of methylene blue than the control group, indicating that the control group had more significant microbial activity and, consequently, more significant feed digestibility. However, there is still no evidence that GAA has an inhibitory effect on the microbiota, as Li et al. [2] showed that there was a linear improvement in the rumen microbiota according to the increased inclusion of GAA in the diet.

SCFAs are produced in the rumen through the degradation of feed by bacteria present in the rumen microbiota [31]. The primary SCFAs are acetic, propionic, and butyric acid and are essential in metabolic processes as a source of energy, synthesis of fats and glucose production. The composition of SCFAs vary according to the composition of the diet [1]. Because SCFA is a product of feed degradation by ruminal bacteria, it is expected that the group that obtained higher MBRT results would present higher levels of SCFAs. In the present study, the control group that obtained shorter MBRT time presented lower levels of total SCFAs, showing that despite containing lower bacterial activity, the bacteria present in the rumen flora of the groups that consumed the additive were more efficient in the production of SCFA. This is different from the results found by Li et al. [2], where cattle in the control group and that supplemented with 0.3 g/kg of dry matter obtained higher levels of acetate and consequently a higher acetate/propionate ratio. In contrast, the groups that received 0.6 and 0.9 g/kg of dry matter had higher levels of propionate and total SCFAs, corroborating our results in which the groups that received GAA had higher SCFA levels. By supplementing Holstein dairy cows with different doses of GAA, Liu et al. [8] showed that the additive increased the level of propionate in the animals’ rumen fluid, corroborating other study [2].

The composition of the meat is one of the most critical aspects for a consumer when purchasing meat, and the growth process is a factor that can affect the quality of the carcass and influences the chemical and physical composition, sex, breed, and nutrition. These are some of the factors that influence the characteristics of the meat. So far, there is a relationship between GAA and the fat content of meat. Still, it is known that one of the effects of creatine supplementation is the increase in intramuscular water retention since creatine attracts water to the muscle, increasing the volume of water in muscle tissue [25].

Beef has a high content of fatty acids and a low ratio between polyunsaturated fatty acids and saturated fatty acids. This difference is due to the biohydrogenation process that occurs in the rumen through the action of various microorganisms. Several factors can affect the composition of fatty acids of meat that varies according to the animal’s diet, where animals with a concentrate-based diet have higher levels of omega-6 fatty acids, and lower levels of omega-3, thus increasing the omega-6/omega-3 ratio [32,33]. Consumers are increasingly looking for foods with the appropriate fatty acid profile since excesses of certain acids, such as saturated acids, can harm human health. Although the groups that received the additive had higher levels of myristoleic, linoleic, and arachidonic acids, there is no relationship between the additive and the higher levels of the acids, mainly because the composition of the fatty acid profile is more linked to the animal’s diet. Omegas are fatty acids with benefits for human health since, if consumed in small quantities, they can cause dermatitis, renal hypertension, type 2 diabetes, arthritis, depression, and decreased resistance to infections, among many other disorders. They are even used as therapy for COVID-19, since omega-3 fatty acids are regulators of some cells of the innate immune system, such as neutrophils, macrophages, basophils and eosinophils [34].

In summary, we found that the research results on GAA in ruminants are similar to what was observed in this study. In another recent study, researchers found that both 0.8 g/kg and 1.6 g/kg of GAA promoted creatine synthesis, nutrient digestibility, and the nitrogen retention ratio, which, in turn, improved growth performance in Angus steers and also showed some antioxidant potential [35]. This oxidative status relationship, according to researchers, is because creatine has been reported to have the ability to remove O^2−^, and the changes in creatine concentration in this study coincided with the changes in antioxidant indices [35,36]. Another recent study showed that GAA has a practical arginine-sparing effect in beef cows during late gestation, in addition to enhancing placental vascularization, which is beneficial at this stage [37], vascularization, which may also be occurring in other organs, as well as being related to differences in blood variables, which deserves future investigation to understand the mechanism. When tested in lambs, researchers concluded that the application of GAA as a feed additive has the bright application prospects of reducing oxidative stress and providing energy to support rumen fermentation in rapid growth [38]. Another interesting finding was that the addition of 0.8% GAA enhanced in vitro rumen fermentation parameters, increased the relative abundance of *Prevotella* and *Prevotellaceae*_UCG-001 in the rumen, and increased the metabolic pathways of bile secretion and protein digestion and absorption [10]. Therefore, it is clear that GAA is a recent additive on the market, which has the potential to improve the productivity of ruminants according to the literature and current studies. The pathways of action of GAA in the body, which are capable of modulating animal nutrition, have been elucidated as well as health and non-metabolic pathways.

## 5. Conclusions

GAA consumed by cattle reduces fat between muscle fibers and serum cholesterol. It has a mild but positive effect on the fatty acid profile of meat; that is, it increases myristoleic, linoleic, and arachidonic acids. We also concluded that GAA consumption increases the amount of volatile fatty acids in the rumen, especially acetic, propionic, and butyric acid. We draw attention to the acetic acid related to lipid synthesis. These findings suggest that GAA supplementation can improve meat quality, potentially increasing economic returns for cattle producers.

## Figures and Tables

**Table 2:** Growth performance of Holstein cattle fed guanidinoacetic acid additive.

Variables	T-0 ^1^	T-3.3	T-6.6	T-9.9	SEM	*p*-Val.: Treat	*p*-Val.: Treat × Day
Body weight (kg)						0.64	0.40
d1	409	412	410	408	5.62		
d15	426	437	427	426	5.71		
d30	455	459	454	455	5.64		
d60	490	492	489	501	5.82		
Weight gain (kg)							
d1–60	81 ^b^	80 ^b^	79 ^b^	93 ^a^	3.17	0.07	-
d15–60	64 ^b^	55 ^c^	62 ^bc^	75 ^a^	2.76	0.01	-
ADG (kg) ^2^							
d15–60	1.42 ^b^	1.22 ^c^	1.37 ^bc^	1.66 ^a^	0.05	0.01	-
ADI (kg) ^2^							
d15–60	11.3	10.9	11.0	11.0	0.14	0.94	-
FC (kg/kg) ^2^							
d15–60	7.99 ^a^	8.96 ^a^	8.00 ^a^	6.63 ^b^	0.25	0.01	-
FE (kg/kg) ^2^							
d15–60	0.125 ^b^	0.111 ^b^	0.124 ^b^	0.150 ^a^	0.006	0.01	-

^1^ Treatments were as follows: T-0, basal diet; T-3.3, feed with the addition of 3.3 g of product/animal/day; T-6.6, feed with the addition of 6.6 g of product/animal/day; T-9.9, feed with the addition of 9.9 g of product/animal/day. ^2^ average daily gain (ADG), average daily intake (ADI), feed conversion (FC), feed efficiency (FE). ^a–c^ Within a row, differ (*p* ≤ 0.05) or tend to differ (*p* ≤ 0.10) between groups.

**Table 3:** Complete blood counts of Holstein cattle fed with guanidinoacetic acid additive.

Variables	T-0 ^1^	T-3.3	T-6.6	T-9.9	SEM	*p*-Val.: Treat ^1^	*p*-Val.: Treat × Day ^1^	*p*-Val.: Day ^2^
Leukocytes (×10^3^/µL)						0.01	0.01	0.01
d1	11.9	10.3	11.0 ^X^	13.2 ^X^	0.65			
d30	10.4 ^a^	9.17 ^ab^	7.69 ^bY^	8.21 ^bY^	0.35			
d60	13.8 ^a^	9.82 ^b^	5.24 ^cY^	6.73 ^cY^	0.34			
Lymphocytes (×10^3^/µL)						0.01	0.01	0.01
d1	7.72	7.46 ^X^	8.30 ^X^	9.57 ^X^	0.62			
d30	6.72 ^a^	6.11 ^abXY^	5.25 ^bY^	4.95 ^bY^	0.32			
d60	6.77 ^a^	4.7 ^bY^	3.22 ^bZ^	3.56 ^bY^	0.32			
Granulocytes (×10^3^/µL)						0.01	0.01	0.15
d1	2.76	1.93	1.66	2.33	0.24			
d30	2.24 ^a^	1.96 ^ab^	1.58 ^b^	2.04 ^ab^	0.15			
d60	4.67 ^a^	3.87 ^a^	1.33 ^b^	2.18 ^b^	0.15			
Monocytes (×10^3^/µL)	1.49	1.09	0.87	1.18	0.09	0.16	0.11	0.62
Erythrocytes (×10^6^/µL)	7.86	7.23	7.23	7.4	0.28	0.90	0.86	0.95
Hemoglobin (mg/dL)	12.0	11.5	11.5	11.3	0.35	0.91	0.82	0.94
Hematocrit (%)	32.1	31.7	31.8	31.6	0.94	0.93	0.87	0.88

^1^ The treatments were as follows: T-0, basal diet; T-3.3, feed with the addition of 3.3 g of product/animal/day; T-6.6, feed with the addition of 6.6 g of product/animal/day; T-9.9, feed with the addition of 9.9 g of product/animal/day. ^2^ Day 1 results were removed from the data set to generate the average per treatment in statistical analysis. ^1^ Within a line (a–c), they differ (*p* ≤ 0.05) between groups for the treatment effect and treatment × day interaction. ^2^ day effect: difference was illustrated by capital letters (X, Y, Z) in the same column and by treatment in each variable.

**Table 4:** Serum biochemistry of Holstein cattle fed with guanidinoacetic acid additive.

Variables	T-0 ^1^	T-3.3	T-6.6	T-9.9	SEM	*p*-Val.: Treat ^1^	*p*-Val.: Treat × Day ^1^	*p*-Val.: Day ^2^
Cholesterol (mg/dL)						0.01	0.01	0.11
d1	89.0	90.3	75.2	75.0	3.54			
d30	102 ^a^	87.5 ^ab^	72.0 ^c^	77.8 ^bc^	3.17			
d60	104 ^a^	96.5 ^ab^	86.3 ^bc^	76.0 ^c^	3.26			
AST (U/L)	64.3	63	54.6	61.8	3.10	0.35	0.28	0.78
GGT (U/L)	18.2	22.6	19.6	18.8	2.57	0.42	0.30	0.91
Total protein (g/dL)	7.10	5.94	6.03	6.14	0.51	0.18	0.12	0.86
Albumin (g/dL)	3.00	2.48	2.75	2.57	0.25	0.13	0.35	0.27
Globulin (g/dL)	4.1	3.45	3.28	3.57	0.26	0.56	0.49	0.52
Urea (mg/dL)	22.8	24.7	21.9	21.3	0.18	0.80	0.72	0.67

^1^ The treatments were as follows: T-0, basal diet; T-3.3, feed with the addition of 3.3 g of product/animal/day; T-6.6, feed with the addition of 6.6 g of product/animal/day; T-9.9, feed with the addition of 9.9 g of product/animal/day. ^2^ Day 1 results were removed from the data set to generate the average per treatment in statistical analysis. ^1^ Within a line (a–c), they differ (*p* ≤ 0.05) between groups for the treatment effect and treatment × day interaction. ^2^ does not have a day effect.

**Table 6:** Carcass, chemical composition, pH, color, water retention capacity, and oxidative status of meat from Holstein steers fed with additive guanidinoacetic acid.

	T-0 ^1^	T-3.3	T-6.6	T-9.9	SEM	*p*-Val.: Treat
Carcass yield (%)	48.4	47.6	46.3	47.4	0.50	0.92
pH—hot carcass	6.55	6.66	6.52	6.58	0.02	0.90
pH—cold carcass	5.47	5.48	5.45	5.45	0.01	0.95
Luminosity	38.7	38.0	38.0	40.6	0.46	0.12
Red color “a”	16.6	17.2	17.0	15.0	0.35	0.24
Yellow color “b”	12.7	13.3	12.5	11.3	0.41	0.38
Retention capacity (%)	80.4	79.0	78.6	80.3	1.32	0.86
Fat (g/kg)	25.3 ^a^	26.5 ^a^	19.3 ^b^	18.5 ^b^	0.74	0.01
Protein (g/kg)	227	224	225	224	1.52	0.96
Organic matter (g/kg)	27.1	26.0	43.0	31.1	4.62	0.15
TBARS (nmol MDS/mL)	18.8	21.2	18.3	22.7	0.96	0.31
Total thiols (nmol SH/mg protiein)	78.3	68.4	78.8	80.7	2.03	0.49
GST (µmol CDNB/min/mg)	756	743	770	774	6.85	0.21

^1^ The treatments were as follows: T-0, basal diet; T-3.3, feed with the addition of 3.3 g of product/animal/day; T-6.6, feed with the addition of 6.6 g of product/animal/day; T-9.9, feed with the addition of 9.9 g of product/animal/day. ^a,b^ Within a row, they differ (*p* ≤ 0.05) or tend to differ (*p* ≤ 0.10) between groups.

**Table 7:** Profile of fatty acids in the meat of Holstein cattle fed with the additive guanidinoacetic acid.

Fatty Acids (g/kg)	T-0 ^1^	T-3.3	T-6.6	T-9.9	SEM	*p*-Val.: Treat
C10:0 (Capric)	0.27	0.24	0.26	0.25	0.03	0.91
C12:0 (Lauric)	0.41	0.41	0.45	0.43	0.06	0.84
C14:0 (Myristic)	19.7	20.2	19.1	20.3	0.78	0.89
C14:1 (Myristoleic)	4.60 ^ab^	3.90 ^b^	5.07 ^a^	5.06 ^a^	0.47	0.05
C15:0 (Pentadecanoic)	2.69	3.02	3.02	2.95	0.33	0.92
C16:0 (Palmitic)	300	304	314	298	2.55	0.16
C16:1 (Palmitoleic)	29.4	34.0	30.8	31.7	0.55	0.12
C17:0 (Heptadecanoic)	7.67	7.63	7.87	8.18	0.84	0.87
C18:0 (Stearic)	160	154	150	153	2.87	0.56
C18:1 n9c (Oleic)	407	399	406	399	6.93	0.83
C18:2n6c (Linoleic)	49.0 ^ab^	52.4 ^ab^	44.53 ^b^	57.51 ^a^	3.42	0.06
C20:0 (Arachidic)	1.15	1.18	1.04	1.12	0.19	0.95
C18:3n6 (?-Linolenic)	0.55	0.80	0.58	0.79	0.05	0.70
C20:1n9 (cis-11-Eicosenoic)	1.43	1.23	1.38	1.37	0.37	0.52
C18:3n3 (a-Linolenic)	1.48	1.58	1.10	1.69	0.44	0.69
C20:2 (cis-11.14-Eicosadienoic)	0.70	0.70	0.56	0.73	0.15	0.20
C22:0 (Behenic)	0.41	0.53	0.37	0.46	0.14	0.58
C20:3n6 (cis-8.11.14-Eicosatrienoic)	3.39	3.42	2.06	3.96	0.75	0.45
C22:1n9 (Erucic)	0.18	0.55	0.32	0.40	0.15	0.11
C20:4n6 (Arachidonic)	7.34 ^b^	8.96 ^ab^	9.00 ^ab^	11.04 ^a^	0.60	0.01
C22:2 (cis-13.16-Docosadienoic)	0.10	0.09	0.06	0.11	0.04	0.94
C24:0 (Lignoceric)	0.37	0.47	0.37	0.45	0.11	0.92
C20:5n3 (cis-5.8.11.14.17-Eicosapentaenoic)	0.13	0.14	0.10	0.20	0.07	0.55
C24:1n9 (Nervonic)	0.15	0.20	0.16	0.19	0.05	0.90
C22:6n3 (cis-4.7.10.13.16.19-Docosahexaenoic)	0.10	0.12	0.00	0.15	0.12	0.38
SFA sum	493.69	492.26	497.52	485.91	6.21	0.16
AGMI sum	443.46	439.52	444.49	437.92	5.12	0.82
AGPI sum	62.85 ^b^	68.22 ^ab^	57.98 ^b^	76.18 ^a^	1.75	0.01

^1^ The treatments were as follows: T-0, basal diet; T-3.3, feed with the addition of 3.3 g of product/animal/day; T-6.6, feed with the addition of 6.6 g of product/animal/day; T-9.9, feed with the addition of 9.9 g of product/animal/day. ^a,b^ Within a row, they differ (*p* ≤ 0.05) or tend to differ (*p* ≤ 0.10) between groups.

## Data Availability

Raw data are held by the authors and may be available upon request.

## Supplementary Materials

The following supporting information can be downloaded at: https://www.mdpi.com/article/10.3390/ani14172617/s1. Supplementary Material S1: Standardization of measurements of volatile fatty acids in bovine rumen liquid.
