# Supplementary material for: Effects of Dietary Guanidinoacetic Acid on the Performance, Rumen Fermentation, Metabolism, and Meat of Confined Steers
Source: Animals (Basel). 2024 Sep 9;14(17):2617. doi: 10.3390/ani14172617 (PMC11394018; doi:10.3390/ani14172617)
Supplement: Supplementary file 1 [file animals-14-02617-s001.zip › animals-3162406-supplementary.pdf]

Supplementary material S1: Standardization of measurements of volatile fatty acids in bovine rumen liquid

Itens	Acetic acid	Propionic acid	Butiric acid	Isovaleric acid	Valeric acid
R2	0.9996	0.9993	0.9983	0.9992	0.9991
Equation	y = 0.0119x + 0.0049	y = 0.0205x - 0.0027	y = 0.026x + 0.00007	y = 0.0319x - 0.0018	y = 0.028x - 0.0009
Linear range (mmol L^{-1})*	4.30 - 128.88	1.69 - 67.63	1.31 - 52.42	0.28 - 13.49	0.28 - 13.58
LOD (mmol L^{-1})	1.07	0.85	0.66	0.28	0.28
LOQ (mmol L^{-1})	2.15	1.69	1.31	0.56	0.57
Accuracy (%)	98.14	93.79	91.16	90.63	99.65
Repeatability (RSD)	4.67	4.52	3.17	2.51	6.79

* The linear range. LOD (limit of detection) and LOQ (limit of quantitation) were expressed in mmol of SFA for L of ruminal fluid
